# A Comparison of Cost-Effective Sensors for a Fluorescence-Based Detection System Used in Biodiagnostic Devices

**DOI:** 10.3390/bios16050276

**Published:** 2026-05-11

**Authors:** Rebecca Vornweg, Christian Decool, Moritz Hemmer, Bastian Stollfuss, Thomas Guenther, Ann-Kathrin Löffler, Roland E. Kontermann, André Zimmermann

**Affiliations:** 1 Institute for Micro Integration (IFM), University of Stuttgart, Allmandring 9B, 70569 Stuttgart, Germany; st191784@stud.uni-stuttgart.de (C.D.); st162077@stud.uni-stuttgart.de (M.H.); bastian.stollfuss@ito.uni-stuttgart.de (B.S.); thomas.guenther@ifm.uni-stuttgart.de (T.G.); andre.zimmermann@ifm.uni-stuttgart.de (A.Z.); 2 Institute for Applied Optics (ITO), University of Stuttgart, Pfaffenwaldring 9, 70569 Stuttgart, Germany; 3 Hahn-Schickard, Allmandring 9B, 70569 Stuttgart, Germany; 4 Institute of Cell Biology and Immunology (IZI), University of Stuttgart, Allmandring 31, 70569 Stuttgart, Germany; ann-kathrin.loeffler@izi.uni-stuttgart.de (A.-K.L.); roland.kontermann@izi.uni-stuttgart.de (R.E.K.)

**Keywords:** cost-effective optical sensors, fluorescence-based diagnostic devices, metal-enhanced fluorescence (MEF), photoresistor, phototransistor, multi-pixel photon counter (MPPC)

## Abstract

Cost-effective, fluorescence-based biodiagnostic devices have the potential to expand point-of-care (POC) testing in resource-limited settings, thereby creating new opportunities for accessible and decentralised diagnostics. The objective of this study is to investigate the performance of a newly designed and simplified system that uses several cost-effective sensors for use in a fluorescence-based biodiagnostic setup. A collecting setup that includes an elliptical mirror to focus emitted fluorescence onto the semiconductor sensors was designed for an intensity-based readout. The readout was realised by various photodiodes, photoresistors, phototransistors and one multi-pixel photon counter (MPPC). Over a range of fluorophore concentrations using serial dilutions of Rhodamine B (RhB), the limit of detection (LOD) and limit of quantification (LOQ) at system level were evaluated. With the exception of the photodiodes, the system demonstrated promising performance with all sensors. The system using the photoresistors achieved the lowest LOD but showed limited repeatability, while the system using the MPPC and phototransistors exhibited high repeatability. A proof-of-concept demonstrated the feasibility of a photoresistor-based configuration by using a sandwich immunoassay for the detection of the antigen Human Epidermal growth factor Receptor 2 (HER2) (100 nM). While this study has not yet encompassed the full spectrum of clinically relevant concentrations, it has yielded valuable insights to enable further enhancements, without the necessity for highly specialised or costly equipment. The limitations and necessary improvements for further developments are discussed.

## 1. Introduction

In order to assess the worldwide rise in emerging infectious diseases [1] and antimicrobial resistance [2], current research focuses on point-of-care (POC) tests with the objective of expanding biodiagnostic tests to pharmacies or to resource-limited settings. Fluorescence-based assays, such as the sandwich immunoassay, are a key component of clinical diagnostics, where fluorophores are used to label detection antibodies [3]. Moreover, physical techniques exist to enhance the fluorescence intensity such as metal-enhanced fluorescence (MEF) [4,5,6] and directing the emission via surface plasmon-coupled emission (SPCE) with high efficiency [5,7,8,9]. The relevant concentration ranges of these biomolecules often vary considerably, necessitating the use of effective detection methods to encompass these concentration ranges [10,11]. Current devices that are already available on the market, in conjunction with biodiagnostic tools and research products, have the capacity to detect low concentrations of biomolecules from µg/mL up to ag/mL [12,13]. However, current devices such as microplate readers are costly, bulky, and inadequately suited for external testing environments. In a variety of experimental setups that use MEF, surface plasmon resonance (SPR) or SPCE, a considerable number of relatively expensive components are used, including lasers [14], spectrometers [15] and charge-coupled devices (CCDs) [16]. These approaches are generally implemented in conjunction with sophisticated optical systems, incorporating excitation and emission filters, lenses, and meticulously arranged optical pathways [16]. When developing POC devices, it is essential to prioritise a simplified overall system architecture and the use of cost-effective components, with the detector being one of the most critical elements. A substantial proportion of the literature focuses on miniaturised or high-performance devices, where complementary metal-oxide semiconductor (CMOS) [17,18] and CCD [16,19,20,21] devices are frequently utilised. In certain instances, photomultiplier tubes (PMTs) [12,21,22] and spectrometers [20,23] are employed in miniaturised setups. In their study, S. Bhaskar and S. S. Ramamurthy presented a smartphone-based SPCE platform [24].

Nevertheless, optical detectors such as photodiodes, photoresistors, phototransistors and multi-pixel photon counters (MPPC) offer a cost-effective and low-complexity solution for fluorescence detection and are rarely used in current devices, particularly as a standalone sensor without an array [25]. The integration of these sensors into an entire POC system and the investigation of their performance at the system level for comparison of suitable cost-effective sensor classes for portable fluorescence-based devices remains an unanswered question. The aim of this study is to address this gap in the current literature by investigating the performance of a newly designed and simplified system that includes cost-effective sensors for use in a fluorescence-based biodiagnostic setup. The proposed system offers a simple, cost-effective and robust alternative for future POC devices by reducing the number and complexity of optical components and utilising user-friendly optical detectors. In contrast to conventional fluorescence detection methods that rely on sensor arrays or camera-based imaging systems [25], this work presents a streamlined architecture that utilises a single light sensor for fluorescence intensity measurement. This reduction in sensing elements leads to a substantially less complex optical and electronic setup. A collecting setup that uses an elliptical mirror to focus emitted fluorescence onto the semiconductor sensors was designed for an intensity-based readout. This was realised by various photodiodes, photoresistors, and phototransistors and one multi-pixel photon counter (MPPC). The performance of the entire system, including the processes of sample preparation and the positioning of each sensor, was investigated. This provides a more realistic representation of how the system would operate in a future POC device. The selection of these sensors was made according to a set of predetermined criteria, with particular emphasis placed on factors such as the sensitivity, photocurrent and wavelength of peak sensitivity, which are of paramount importance for the present application. The minimum detectable signal (MDS) on the basis of the root mean square (RMS) and the limits of detection and quantification (LOQ) at the system level were assessed across a range of fluorophore concentrations. To ensure sufficient signal intensity, MEF was exploited, where fluorophores in close proximity to a metallic surface exhibit enhanced emission, and SPCE was employed to direct fluorescence towards the detector. In SPCE sensors, recent decades have seen extensive investigation of angular shifts upon analyte binding, with the potential to achieve high sensitivities [13]. In this study, the intensity variations in the emitted fluorescence, which depends on the analyte concentration, were measured directly with each sensor. This approach replaced angular scanning, which is typically used to monitor SPCE shifts [21,26]. The elliptical mirror in the provided system collects emitted fluorescence and focuses it onto the detector, while a thin gold coating demonstrates the feasibility in MEF and SPCE applications. A preliminary proof-of-concept using a sandwich immunoassay with a fluorophore-labelled secondary antibody illustrates biological applicability, although detailed assay optimisation lies beyond the scope of this study. The findings provide guidance for selecting appropriate cost-effective optical detectors for qualitative versus quantitative analysis in the system presented here. In conclusion, this work establishes a fundamental basis for future developments of a POC device aimed at achieving clinically relevant detection ranges.

## 2. Materials and Methods

**Figure 1:**
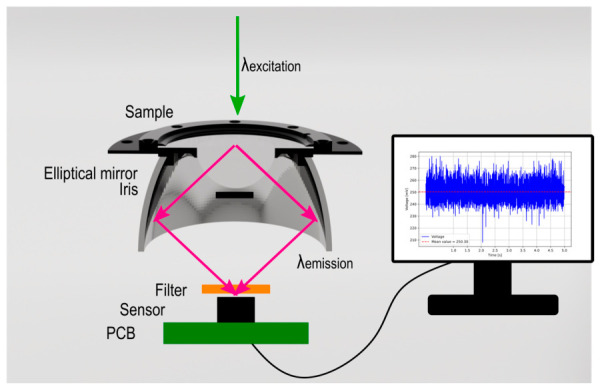
Measurement setup for efficient collection and focusing of fluorescent light with λ_emission_. The samples were placed on the glass slide by using immersion oil for the purpose of optical coupling and were illuminated by the laser with λ_excitation_ from above. An iris composed of light-absorbing material was integrated into the optical pathway to block the main light of the excitation. A hard-coated long-pass filter was placed directly in front of the detector to further minimise the detection of the excitation light.

The aim of the newly designed and simplified system presented here was to focus the emitted fluorescent light in order to enhance the fluorescence intensity while simultaneously separating the excitation light from the fluorescent emission in order to reduce the overlap between the excitation and the emission signals. For this purpose, a measurement configuration was devised and is illustrated in Figure 1. The primary optical component of the apparatus is an elliptical mirror (#90-973 from Edmund Optics, Barrington, NJ, USA), which collects and focuses the emitted fluorescence from the bottom of the sample to the second focus point. The configuration of the apparatus is such that the bottom of the substrate, from which the fluorescence is emitted, is aligned with the first focus point. The second focus point is situated beneath the elliptical mirror, where the detection process was conducted. In order to circumvent total internal reflection or refraction of the light passing through the substrate into the air, a lens (LA1252 from Thorlabs, Newton, NJ, USA) was integrated into the sample stage. The glass plate installed on the upper surface of the lens serves both functional and aesthetic purposes: it protects the lens from scratches and dirt, as well as ensuring ease of cleaning. In order to facilitate the optical coupling between the glass and the lens, an immersion oil was used between the two components. An iris composed of light-absorbing material was integrated into the optical pathway to block the main light of the excitation. In order to further minimise the detection of the excitation light, a hard-coated long-pass filter (FELH0600 from Thorlabs, Newton, NJ, USA) was placed directly in front of the detector. With the exception of the long-pass filter, all components are to be fixed in such a manner that the sample stage constitutes a completely connected part. The samples were then placed on the surface of the glass plate.

In accordance with the specified requirements, two photoresistors (Advanced Photonix, Camarillo, CA, USA), two photodiodes (SFH2711A01 from OSRAM, Munich, Germany; BPW21R from Vishay Intertechnology, Malvern, PA, USA), two phototransistors (OSRAM, Munich, Germany) and one MPPC (Hamamatsu Photonics K.K., Hamamatsu, Japan) were selected for the purpose of intensity-based readout. The following section provides an overview of the sensors evaluated in this study. The overview includes the specific product name, its sensitivity, its price, and the resistance values used during the measurements, which are stated in Table 1. The ascent and descent times, as well as the light and dark resistance, can be found in respective data sheets [27,28]. Both photoresistors have an ascent time of 60 ms and a descent time of 25 ms. PDV-P9008 exhibits higher resistance values (R_D_ = 20 MΩ and R_L_ = 10–200 kΩ) in comparison to PDV-P9004 (R_D_ = 2 MΩ and R_L_ = 27–60 kΩ).

**Figure 2:**
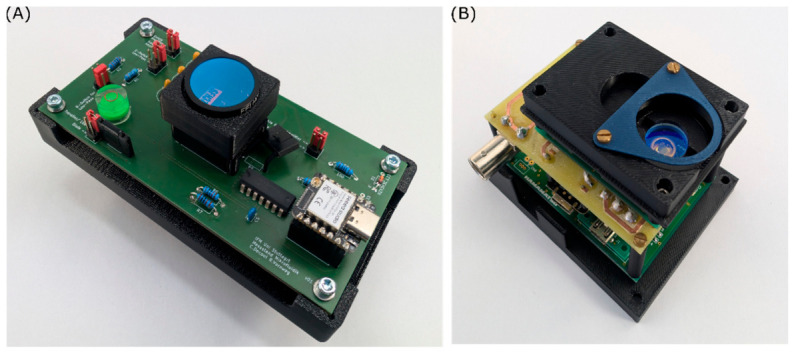
(**A**) PCB for the integration of the phototransistors, photodiodes, and photoresistors. (**B**) MPPC with supplementary PCB for voltage supply and evaluation board (**right**). The long-pass filters are installed above the sensors.

The photoresistor was utilised as a voltage divider, where the voltage measured across the fixed resistor is indicative of the light intensity. The photodiode was operated in photovoltaic mode (zero bias), a condition that is characterised by the absence of a pre-voltage, with the objective of suppressing the dark current. The phototransistor was operated as a simple photodetector. All circuits are integrated on a single printed circuit board (PCB), shown in Figure 2A, with the presence of a common evaluation circuit to facilitate handling and to minimise the influence of rebuilding on the output. Additionally, two resistors and three capacitors for an RC circuit were incorporated to enable filtering of the analogue voltage signal of each sensor, thereby ensuring that a minimum cut-off frequency of approximately 100 Hz could be attained. The operational amplifier (OA) LM324N (Texas Instruments, Dallas, TX, USA) was utilised to amplify and convert the impedance of the sensor signal. The integrated circuit package contains four independent OAs that are designed to operate with a single supply voltage and have a maximum input bias current of 50 nA. The analogue signals were detected by a microcontroller (XIAO ESP32S3 μC, Seeed Studio, Shenzhen, China) with an operating voltage of 3.3 V, which contains a 12-bit analogue-to-digital converter on a single chip. The microcontroller enables the detection of voltage changes of 0.805 mV, and is connected to a computer.

The MPPC is not included on the PCB, as it is equipped with its own board C14450 (Hamamatsu Photonics K.K., Hamamatsu, Japan). A supplementary PCB was conceptualised to enable the voltage supply of ±5 V, with a maximum deviation of ±0.25 V, encompassing filtering, smoothing and evaluation function. The MPPC is shown in Figure 2B. The evaluation of the sensor signal is conducted by the same microcontroller as the other sensors.

In order to evaluate the sensor response as a function of the concentration, a series of dilutions of a diluted fluorophore was utilised. In this study, the fluorophore Rhodamine B (RhB) (CAS nr. 81-88-9 with C.I. 45170 from Carl Roth GmbH + Co. KG, Karlsruhe, Germany) was employed as a fluorescent sample. The stock solution was composed of 5 g of polyvinyl alcohol (PVA) (CAS nr. 9002-89-5 from Carl Roth GmbH + Co. KG) for stabilisation of RhB and 0.05 g of RhB, which had been diluted in 500 mL of distilled water. The stock solution (sample 1) was then subjected to a gradual dilution process, whereby the concentration was reduced by half until it reached sample 19. The dilution series is displayed in Table 2. Sample 20 does not contain RhB, thus serving as a blank sample.

For the purpose of sample preparation, glass substrates made of soda–lime glass were used, and underwent a 10 min cleaning procedure in Helmanex III (Hellma GmbH & Co. KG, Müllheim, Germany) employing ultrasound technology. Following the cleaning process, the substrates were coated with 2 nm of titanium, followed by 33 nm of gold by thermal vapour deposition. The choice to employ gold was made due to its biocompatibility and inertness. Afterwards, the substrates were cut into pieces of 10 × 10 mm^2^ and 1 µL of the solution with a specific concentration was applied to each substrate using a 10 µL pipette (EP3123000020, Eppendorf, Hamburg, Germany), which was then covered with a cover slide to avoid drying. In order to avoid total internal reflection between the sample and the sample stage, immersion oil was added to the glass. Subsequently, the samples were inserted on the sample stage.

The absorption maximum of RhB is at the wavelength of 560 nm, and the emission wavelength is at 585 nm. Consequently, the laser (CW532-01C from Roithner Laser Technik GmbH, Vienna, Austria) was used as the excitation source for the fluorophore. In order to minimise unstable illumination effects of the laser, the device was activated 30 min prior to each measurement.

Before each measurement, the glass of the sample stage was cleaned with ethanol. The initial focus point of the elliptical mirror was determined with the sample exhibiting the most pronounced concentration being utilised for this purpose. The detector’s surface of the sensor with the smallest height (SFH2711A01) was installed at the focus point. The height of the other sensors was measured before and positioned in relation to the smallest sensor. It is important to note that none of the sensors was measured twice in succession. The sensors were changed after each series of measurements. In summary, the measurements of each sensor were repeated three times.

Following the completion of the measurement preparation, the measurements were obtained from each sensor. For the initial measurement the blank sample (sample 20) was used. The fixed resistance for each sensor during the measurements was chosen such that the signal of the blank sample approximated zero. These resistor values are outlined in Table 1. The measurements were subsequently conducted from the lowest to the highest RhB concentration (from sample 19 to sample 1). The measurements were conducted within a black box to eliminate the potential impact of ambient light on the results. The signal was queried at a sampling rate of 500 Hz for a period of 5 s by the controller. Mean value and standard deviation were then calculated.

In order to demonstrate the suitability of the developed setup for POC applications, a proof-of-concept measurement was conducted using a sandwich immunoassay. As Trastuzumab, a monoclonal antibody used to treat Human Epidermal growth factor Receptor 2 (HER2)-positive breast and stomach cancer, had already received clinical approval, it was selected for this measurement [36]. HER2-moFc was selected as the antigen to be detected. The presence of HER2 has been identified in breast cancer cases and has the capacity to serve as an indicator of the course or success of treatment [37]. The sandwich immunoassay was performed in which the primary antibody was immobilised in 1× phosphate-buffered saline (PBS) at 4 °C overnight. Remaining nonspecific binding sites were blocked with 2% (*w*/*v*) skim milk in PBS (MPBS). HER2-moFc, used as a proof-of-concept diagnostic marker, was added at a concentration of 100 nM in MPBS and incubated for 1 h at room temperature (RT). Detection was carried out using a phycoerythrin (PE)-labelled murine Fc-specific secondary antibody (59997S from Cell Signaling Technology, Danvers, MA, USA), which was incubated for 1 h at RT. The measurement was conducted for 5 s at a sampling rate of 500 Hz using the photoresistor P9008 with a resistance of 400 kΩ.

## 3. Results

The following results describe the performance of the entire system using various photoresistors, followed by photodiodes, phototransistors and one MPPC, when exposed to samples of different concentrations of RhB. The dashed lines in the graphs indicate the value of the blank sample as a reference.

**Figure 3:**
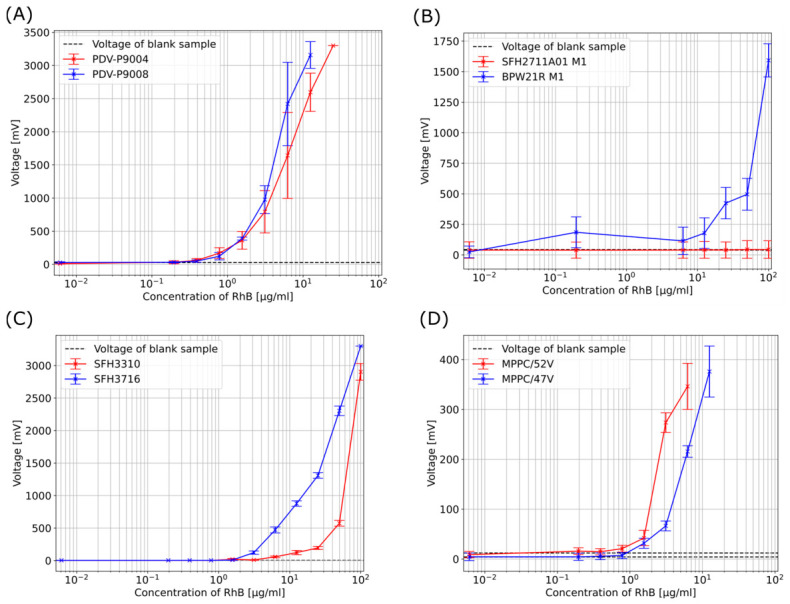
(**A**) The mean system response using two different photoresistor-based configurations, plotted versus a sample concentration, derived from three repeated measurement runs (M1–M3), each using newly prepared samples. (**B**) A comparison of system outputs using two different photodiode-based configurations recorded for multiple sample concentrations. Each curve represents one repeated measurement under identical conditions. (**C**) The mean system response using two different phototransistors plotted versus sample concentration derived from three repeated measurement runs (M1–M3), each using newly prepared samples. (**D**) The mean system response using the MPPC-based configuration, conducted with 47 and 52 V, and plotted versus sample concentration derived from three repeated measurement runs (M1–M3), each using newly prepared samples. In all plots, the dashed line indicates the blank sample value. The measurements for each concentration were conducted for 5 s at a sampling rate of 500 Hz, after which the mean value and standard deviation were calculated. The results incorporate the full system-integrated variability.

Figure 3A presents the performance of the system using the two photoresistors. The relatively high standard deviation for each concentration indicates that the repeated measurement runs (M1 to M3) exhibit noticeable variation. It is evident that the standard deviation increases with an increase in RhB concentration, thus indicating greater measurement variability at higher concentrations. For lower concentrations the photoresistors demonstrate consistent performance up to sample 9 (0.391 µg/mL), which still exhibits slight deviations from the blank sample. In general, both photoresistors demonstrate comparable performance in the entire system. However, photoresistor P9008 exhibits elevated values at elevated concentrations.

As illustrated in Figure 3B, the measurement results using the two photodiodes in the entire system are presented. It was not possible to obtain any measurable values with the photodiode SFH2711A01, even for the highest concentration of RhB. The photodiode BPW21R demonstrates superior performance in comparison to the other photodiode. However, it exhibits irregular values in the case of sample 10 (0.195 µg/mL), where the value was higher than that of sample 5 (6.250 µg/mL). Due to the irregular behaviour of this photodiode and the insufficient performance of the other photodiode the measurements were not repeated.

As demonstrated in Figure 3C, using three phototransistors, the system exhibits high repeatability, resulting in low standard deviation across the concentrations. A minor increase in standard deviation is evident as the concentration rises, indicative of a modest enhancement in measurement variability. Phototransistor SFH3716 consistently yields higher measured values than phototransistor SFH3310 across the concentration range. In the case of sample 6 (3.125 µg/mL), the measured value obtained by phototransistor SFH3716 deviates from the blank value, whereas the corresponding measurement from phototransistor SFH3310 remains as low as the blank value.

Figure 3D presents the measurements using the MPPC in the entire system conducted at two different supply voltages: 47 and 52 V. It is important to note that the *Y*-axis displays a reduced range, which can be attributed to the MPPC’s distinct voltage output compared to the other sensors. The standard deviation for both voltage settings increases with an increase in the concentration of the sample, indicating higher variability at elevated concentrations. A comparison of the system using the MPPC with the system using the other sensors reveals that its standard deviation is comparable to that of the phototransistors, and lower than that of the photoresistors. Across the entire concentration range, the MPPC measurements at 52 V are consistently higher than those at 47 V, including those at lower concentrations. For the 52 V measurements, the mean value of sample 8 (0.781 µg/mL) exhibits a slight deviation from the blank sample, whereas for the 47 V measurements, this deviation first appears at sample 7 (1.563 µg/mL).

For further comparison, the RMS, MDS, LOD, and LOQ of the best-performing system response for each sensor type were analysed. The RMS was derived by calculating the mean value for offset correction, which was then deducted from each individual value. The RMS was then calculated from the corrected values of the blank sample $U_{blank,c}$ and the total number of values n as

(1) $$RMS=\sqrt{\frac{1}{n}(U_{blank,c,1}^{2}+U_{blank,c,2}^{2}+\ldots +U_{blank,c,n}^{2})}$$

The RMS values of the individual measurement runs were combined to produce a single, pooled RMS value. The MDS is defined as $MDS=3\times {RMS}_{pool}$. The RMS and MDS are shown in Table 3.

For further analysis, the LOD was calculated by the intersection of the four-parameter logistic (4PL). The signal threshold $y_{LOD}$ was defined by the blank signal $\bar{U_{blank}}$ and its standard deviation $\sigma_{blank}$, i.e.,

(2) $$y_{LOD}=\bar{U_{blank}}+3.29\sigma_{blank}.$$

Given that the standard deviation of the low-level samples was found to be comparable to that of the blank sample, the simplified Currie criterion for constant variance for lower concentrations was applied [38]. Each measurement dataset was fitted by using the nonlinear-fit function ‘Logistic’ of the software OriginPro 2021. The 4PL is defined by various parameters, where y is the measured signal (voltage in mV), x is the analyte concentration of the samples, $A_{1}$ is the asymptotic minimum, $A_{2}$ is the asymptotic maximum, $x_{0}$ is the inflection point, and p is the Hill slope, determining the steepness of the curve [39].

The LOD was calculated by solving this equation for x as the intersection between $y_{LOD}$ and the fitted function, as outlined in Equation (3), defined by

(3) $$y=\frac{A_{1}-A_{2}}{1+{\left(x/x_{0}\right)}^{p}}+A_{2}$$

The precision of the LOD determination is affected by the variance in the blank measurement and the fitting. The precision regarding fitting was ensured by the goodness-of-fit of the 4PL model, yielding a coefficient of determination R^2^ > 0.99 for the system using the photoresistor and the MPPC. Due to the sharp rise in the phototransistor’s voltage, a slightly lower R^2^ > 0.96 could be achieved during the fitting process. This results in a slight increase in the LOD. The full set of fitted graphs and the corresponding numerical fitting parameters can be found in the supplementary data repository [DOI: https://doi.org/10.18419/DARUS-5825].

**Figure 4:**
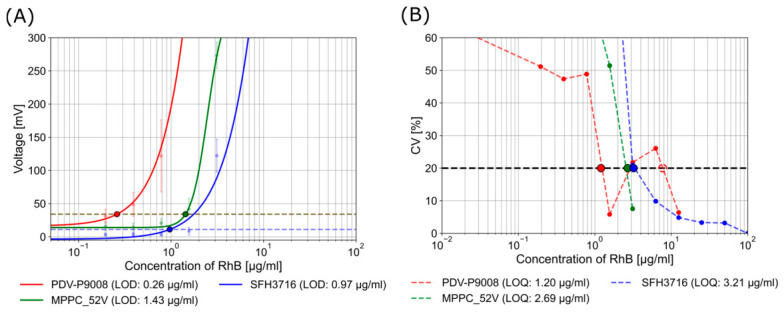
(**A**) The LOD of the mean system response using a configuration with the photoresistor PDV-P9008 (red), the phototransistor SFH3716 (blue) and the MPPC (green) with 52 V derived from three repeated measurement runs, each using newly prepared samples. The dashed lines indicate the LOD. The green and red dashed lines are in close proximity to each other, which results in the red line’s visibility being compromised. (**B**) The CV of the mean sensor response derived from three repeated measurement runs, each using newly prepared samples and an integration time of 5 ms. The dashed line indicates the LOQ as 20%. The results of LOD and LOQ incorporate the full system-integrated variability.

The LOQ was defined as the lowest concentration with a coefficient of variation (CV) CV ≤ 20% [40]. These definitions are based on recommendations from the Clinical and Laboratory Standards Institute (CLSI EP17-A2, 2012) [41] and are consistent with values commonly used in bioanalytical validation studies [42,43]. CV was calculated as

(4) $$CV=\frac{\sigma_{S}}{\bar{U_{S}}-\bar{U_{blank}}}\times 100\%,$$

with the signal variation per concentration playing a decisive role in the calculation. As outlined in Equation (4), the CV was calculated as the quotient of the standard deviation of the sample measurements $\sigma_{s}$, and the corrected mean value of the voltage. The corrected mean value is given by the sample $\bar{U_{S}}$ minus the voltage given by the blank $\bar{U_{blank}}$. The decision to employ the CV-based variant [44] was motivated by the presence of heteroscedasticity. The dashed lines in Figure 4 indicate LOD and LOQ.

Figure 4 demonstrates that elevated sample concentrations result in increased measurement variability. Conversely, the system shows more stable performance at lower concentrations, which is advantageous for rapid testing applications where the detection of minimal concentrations is essential. The LOD using the photoresistor can be defined as ~0.3 µg/mL, while the definition of the LOQ is more challenging to ascertain. It is evident that the variability of the three measurements within the system using the photoresistor has resulted in inconsistent values for the LOQ. This phenomenon is exemplified in the range of 1.2 to 8 µg/mL. In this area, the samples demonstrate fluctuations that coincide with the limits of the LOQ. The analysis of the LOQ using the phototransistor and the MPPC with 52 V is between these concentrations, showing an LOQ of 3.2 µg/mL and 2.7 µg/mL, respectively. However, using the photoresistor, the system displays comparatively low CV values at lower concentrations, when compared with the system using the phototransistor and MPPC. In comparison to the photoresistor, the system with the MPPC and the phototransistor demonstrates a reduced LOD of ~1.4 µg/mL and ~1.0 µg/mL, respectively.

**Figure 5:**
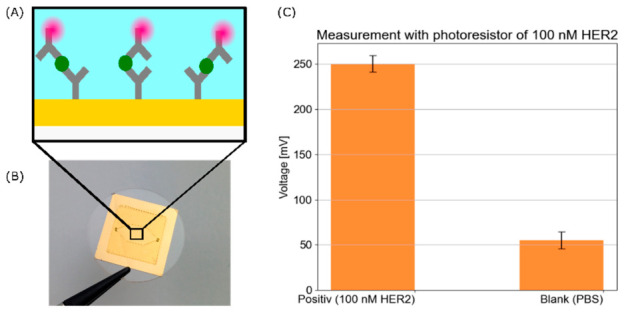
Proof-of-concept measurement of a sandwich immunoassay for antigen detection using a fluorescently labelled secondary antibody. (**A**) The schematic of the sandwich immunoassay, comprising the primary antibody Trastuzumab, the target antigen HER2-moFc (green), and the PE-labelled murine Fc-specific secondary antibody (red). (**B**) The microfluidic chip used for the assay. The same substrate as for the other measurements was used. The chip is covered by the polymer COP690R. (**C**) The measurement results obtained using the setup described above with photoresistor P9008 with a resistance of 400 kΩ. The antigen concentration was 100 nM. The measurement was conducted for 5 s at a sampling rate of 500 Hz, and the mean value and standard deviation were subsequently calculated.

The following results demonstrate the proof-of-concept measurement, which was conducted using a sandwich-immunoassay and the photoresistor P9008. The employed sandwich immunoassay, in which the primary antibody Trastuzumab was immobilised on a gold surface, followed by the target antigen HER2-moFc at a concentration of 100 nM (≙9.85 µg/mL), and finally the PE-labelled murine Fc-specific secondary antibody, is demonstrated in Figure 5A. A microfluidic chip, shown in Figure 5B, was used for the assay. As demonstrated in Figure 5C, the findings suggest that, under these conditions and employing the same coated substrates, the sensor can detect the antigen at the designated concentration, thereby validating the viability of biomolecule detection within this system. It has to be noted that for the proof-of-concept, only one chip for the positive and one chip for the blank sample were fabricated and measured.

## 4. Discussion

In this study, the performance is evaluated at the system level rather than isolating the sensor as a standalone component. The results obtained are a reflection of the performance of the entire system, including the processes of sample preparation and the positioning of each sensor. This comprehensive approach reflects the current research stage, where the focus is on demonstrating the fundamental feasibility of using such sensors within an integrated analytical setup. This provides a more realistic representation of how the system would operate in a future POC device, which is an important consideration. A rigorous and repeatable methodology was developed with meticulous attention to detail, thereby minimising deviations caused by factors such as sample preparation and system accessory hardware. This is supported by the high degree of repeatability demonstrating by the use of the phototransistors and the MPPC, which indicates that, despite the influences at system level, a high degree of repeatability can be achieved with the sensors selected.

The results presented here are intended to serve as a guide when selecting suitable sensor options for the measurement system presented. The following conclusions are drawn on the basis of three independent measurement runs with freshly prepared samples for each sensor, which is considered sufficient for this approach. A detailed characterisation of a selected, preferred sensor, including a significantly larger number of measurements, is planned for future work. The calculations of LOD and LOQ incorporate the analyte-specific sensitivity of the sensor in the overall system and are summarised in Table 4. It should be noted that the LODs and LOQs are not universal but system-specific, as they depend on the quantum yield of the specific fluorophore and the optoelectronic configuration of the measurement setup.

The lower repeatability using a photoresistor in comparison to using a photodiode or phototransistor in the system is consistent with the findings of other studies [45,46]. The reduced repeatability can be attributed to the extended rise and decay time as a consequence of the photoelectric inertia caused by charge carrier dynamics [47] and the nonlinear behaviour of the photoresistor material [46]. The limited repeatability of measurements with the photoresistors should therefore be examined in future work to determine whether this behaviour is intrinsic to the sensor or a consequence of the measurement configuration.

According to the results of this study the use of a photodiode does not appear to be a viable option for a fluorescence-based test device at this stage, primarily due to its substandard performance resulting from the low photocurrents it generates. In comparison to the photodiode a phototransistor is able to generate higher measurable signals in the setup presented here than a photodiode due to its intrinsic amplification of the light-induced current. This inherent amplification mechanism is absent in a photodiode, resulting in significantly lower overall sensitivity [48]. For further investigations, a special transimpedance amplifier could be used to improve the signal quality of the photodiode. Furthermore, an additional evaluation circuit operating in photoconductive mode could also be included in the testing process. For both the photodiodes and phototransistors examined, the sensor that performed better was the one with a wavelength of maximum sensitivity closer to the wavelength of the fluorescence emission. This indicates the significance of the wavelength at maximum sensitivity when selecting an optical sensor.

In the case that the objective is to differentiate between blank, low, and high concentrations, as opposed to quantifying them, it appears that the photoresistor is the optimal choice for the system presented here. The system using the photoresistor demonstrates a high degree of reliability in detecting low concentrations, achieving the lowest LOD among all tested sensors. Conversely, if the objective is quantification, the system using the MPPC and the phototransistor showed a higher suitability as it demonstrated a higher degree of repeatability across the concentration range. This level of consistency is crucial for producing reproducible and quantitative concentration estimates. The phototransistor demonstrated the lowest RMS in comparison to the other sensor types, as illustrated in Table 3. When using the MPPC it is important to note that only a reduced concentration range can be measured using one supply voltage. However, these advantages are accompanied by a substantially higher cost among the evaluated devices. Furthermore, it cannot be ruled out that shadows may be caused by the housing of the sensor when the angle of incidence of the light is high. These drawbacks may limit its suitability for cost-effective applications. The selection of the most suitable sensor is ultimately contingent upon the intended application and the desired measurement objective. In this context, the relative prioritisation of factors such as achieving the lowest possible LOD, ensuring highly repeatable quantification, or balancing performance and economic considerations must be taken into account. Furthermore, it should be noted that most photoresistors are not yet RoHS-compliant, as they contain cadmium (Cd) or cadmium compounds [49,50].

Additionally, it is important to note that the responsivity of photodetectors can vary depending on the angle of incidence of incoming light. The highest sensitivity for the phototransistors and the photodiodes used in this study is achieved at perpendicular incidence [31,33,34]. Due to the characteristics of the elliptical mirror and the fluorescence emission, the light does not make a perpendicular incidence at the sensor surface. This may reduce the effective irradiance on the phototransistor, resulting in a weaker signal being detected. In plasmonic-based approaches, the angle of emission can vary greatly from approximately 45° to 70° depending on the refractive index of the sample liquid. It is important to note that this angle may also vary slightly with each measurement and during a measurement with the same sample liquid [51]. In order to enhance the viability of the detectors in this configuration, the implementation of a collimation setup is considered a viable option. This issue must be addressed in future work.

When relating the determined LOD and LOQ to potential POC applications, it is important to consider the clinically relevant concentration ranges of the biomolecules intended for detection. Depending on the specific target analyte, clinically significant concentrations can vary widely, typically spanning from pg/mL (fM to pM) to mg/mL (nM to µM). High-abundance antigens demonstrate concentrations within the range of mg/mL like human serum albumin, immunoglobulin G and fibrinogen [10], whereas low-abundance antigens like Tau protein exhibit concentrations of pg/mL [11]. With regard to the subject of tumour markers, the level of healthy patients is situated within the lower range of a few ng/mL [52,53], which can undergo an increase in cases of cancer [54]. Consequently, the concentration range of greatest relevance for POC devices has to cover a wide range of concentrations, depending on which antigen is aimed to be detected. Despite the sensors evaluated in this study not yet encompassing the full spectrum of clinically relevant concentrations for reliable measurement of tumour markers, future work will concentrate on enhancing the LOD and LOQ across different sensor types, e.g., by the integration of high-frequency filters, and improvements to alignment, circuits and shielding. While current state-of-the-art ranges extend from µg/mL down to ag/mL, our platform already competes effectively within the µg/mL range using a significantly simpler and more cost-effective setup [12,13]. As the required LOD varies according to the antigen, achieving extremely low LODs is not always a clinical necessity. Future development will thus balance improved detection limits with the robust and accessible design presented here. One potential solution to the issue of expanding the concentration range, in particular for the photoresistor or phototransistor, is to use resistor networks. The MPPC could be operated at varying voltages to enhance the measuring range.

The proof-of-concept demonstrates the general applicability of the photoresistor as a cost-effective detector for fluorescent biomolecules within the presented setup. Further investigations are required using the sandwich immunoassay to determine the LOD and LOQ of different sensors under biologically relevant conditions. The aim is to enhance the sensor’s LOD towards concentrations lower than 100 nM (≙9.85 µg/mL), thereby approaching clinically meaningful ranges. Therefore, it is essential to consider the impact of real biological samples. Proteins, enzymes, drugs, and other components found in biological fluids may result in cross-reactivity, non-specific binding or signal distortion [55]. In order to ensure reliable performance of the assay, it is necessary to establish strict methods, such as sample pre-treatment and the use of controls.

## 5. Conclusions

The designed setup for this study, using an elliptical mirror to focus emitted fluorescence onto various semiconductor sensors, demonstrated the feasibility of a simplified intensity-based readout over a range of RhB concentrations (from 0.4 ng/µL to 100 µg/mL). With the exception of the photodiodes, the system demonstrated promising performance with all sensors. In order to differentiate between positive and negative samples, a photoresistor could be the optimal choice for the system presented here, as it has achieved the lowest LOD, though its repeatability is limited. A proof-of-concept demonstrated the feasibility of a photoresistor-based configuration by using a sandwich immunoassay for the detection of the antigen HER2 (100 nM). With regard to quantification, the system using the MPPC and phototransistors is more promising, as high repeatability and thus a lower LOQ were exhibited. While this study has not yet encompassed the full spectrum of clinically relevant concentrations, it has yielded valuable insights to achieve further enhancements, without the necessity for highly specialised or costly equipment. Additional studies are required to assess whether the system using these cost-effective sensors can provide quantitative measurements or is limited to qualitative detection. In the event of quantitative performance being achievable, the suitability of the system in combination with the sensors for robust calibration procedures will need to be established.

## Figures and Tables

**Table 1:** Overview of analysed semiconductor sensors. Sensitivity, wavelength at peak sensitivity and photo current are from the respective data sheets. The price for each sensor is based on orders placed in May 2025. The fixed resistance for each sensor during the measurements was chosen so that the signal of the blank sample approximated zero.

Sensor Art	Name	Sensitivity	Wavelength at Peak Sensitivity [nm]	Photo Current [µA]	Price (Gross) [€]	Fixed Resistance During Tests [kΩ]
Photoresistor [27,28,29,30]	PDV-P9008	0.85 Ω/lx	570	n.s.	1.30	400
	PDV-P9004	0.85 Ω/lx	570	n.s.	0.97	200
Photodiode [31,32]	SFH2711A01	0.115 nA/lx	580	0.12 @ 100 lux	0.93	10,000
	BPW21R	9 nA/lx	565	9 @ 1000 lux	10.33	10,000
Phototransistor [33,34]	SFH3310	n.s.	615	450 @ 1000 lux	1.01	300
	SFH3716	n.s.	570	49 @ 100 lux	0.74	300
MPPC [35]	S14420-3050MG	n.s.	600	n.s.	130.00	-

**Table 2:** Concentration of diluted fluorophores in PVA/water solution. The concentrations of samples 1 to 10 are expressed in µg/mL, whereas the concentrations of samples 11 to 20 are expressed in ng/mL.

**Sample**	**1**	**2**	**3**	**4**	**5**	**6**	**7**	**8**	**9**	**10**
Concentration [**µg**/mL]	100.000	50.000	25.000	12.500	6.250	3.125	1.563	0.781	0.391	0.195
**Sample**	**11**	**12**	**13**	**14**	**15**	**16**	**17**	**18**	**19**	**20**
Concentration [**ng**/mL]	97.7	48.8	24.4	12.2	6.1	3.1	1.5	0.8	0.4	0.0

**Table 3:** RMS and MDS of the mean system response, using a configuration with the photoresistor PDV-P9008, the phototransistor SFH3716 and the MPPC with 52 V. The results incorporate the full system-integrated variability, derived from three repeated measurement runs, each using newly prepared samples.

Sensor	RMS [mV]	MDS [mV]
PDV-P9008	6.09	18.26
SFH3716	1.89	5.66
MPPC/52V	6.67	20.04

**Table 4:** The LOD and LOQ of the mean system response, using a configuration with the photoresistor PDV-P9008, the phototransistor SFH3716 and the MPPC with 52 V, derived from three repeated measurement runs, each using newly prepared samples. The results incorporate the full system-integrated variability and are presented with a precision of one decimal place.

Sensor	LOD [µg/mL]	LOQ [µg/mL]
PDV-P9008	0.3	1.2/8.0
SFH3716	1.0	3.2
MPPC/52V	1.4	2.7

## Data Availability

The datasets generated and/or analysed during the current study are available under CC BY-NC-ND 4.0 in DaRUS under DOI: https://doi.org/10.18419/DARUS-5825.

## Abbreviations

The following abbreviations are used in this manuscript:

4PL	Four-parameter logistic
CCD	Charge-coupled device
Cd	Cadmium
CMOS	Complementary metal-oxide semiconductor
CV	Coefficient of variation
HER2	Human Epidermal growth factor Receptor 2
LOD	Limit of detection
LOQ	Limit of quantification
MDS	Minimum detectable signal
MEF	Metal-enhanced fluorescence
MPBS	Skim milk in PBS
MPPC	Multi-pixel photon counter
OA	Operational amplifier
PBS	Phosphate-buffered saline
PCB	Printed circuit board
PMT	Photomultiplier tubes
POC	Point of care
PVA	Polyvinyl alcohol
RhB	Rhodamine B
RMS	Root mean square
RT	Room temperature
SPCE	Surface plasmon-coupled emission
SPR	Surface plasmon resonance
